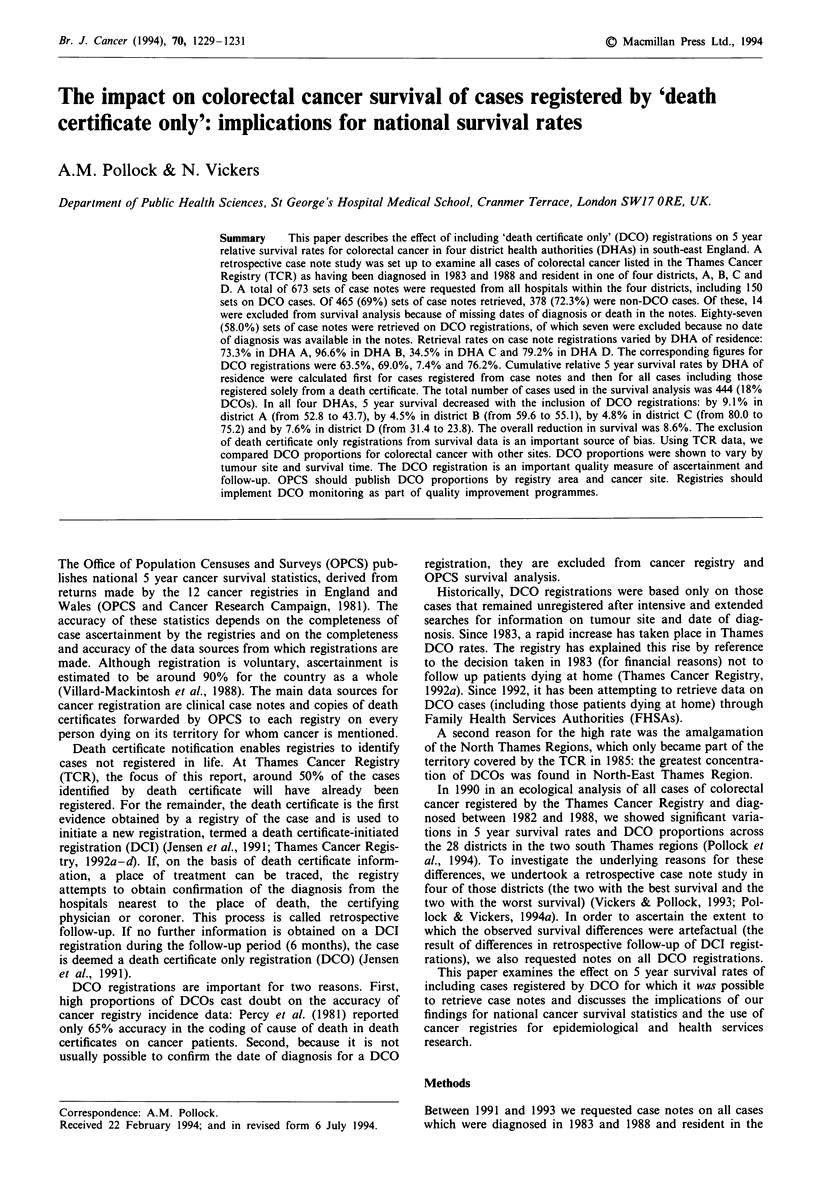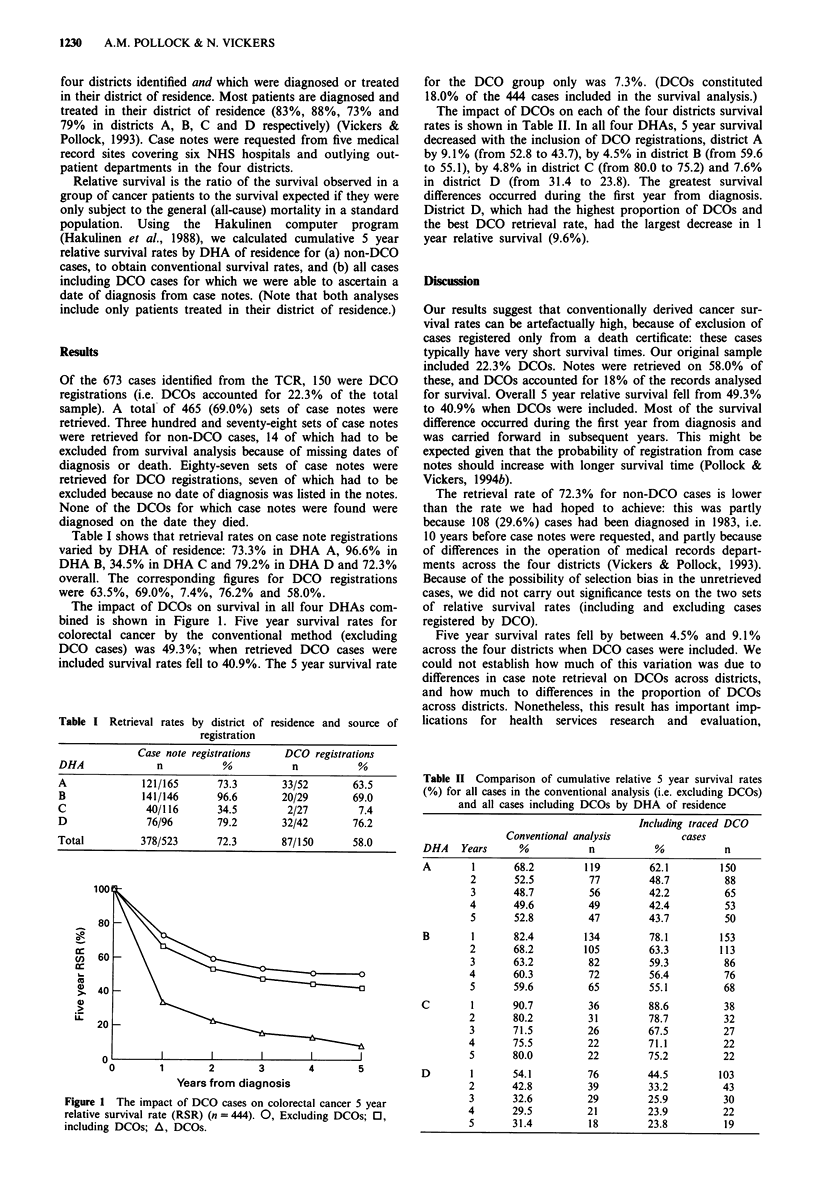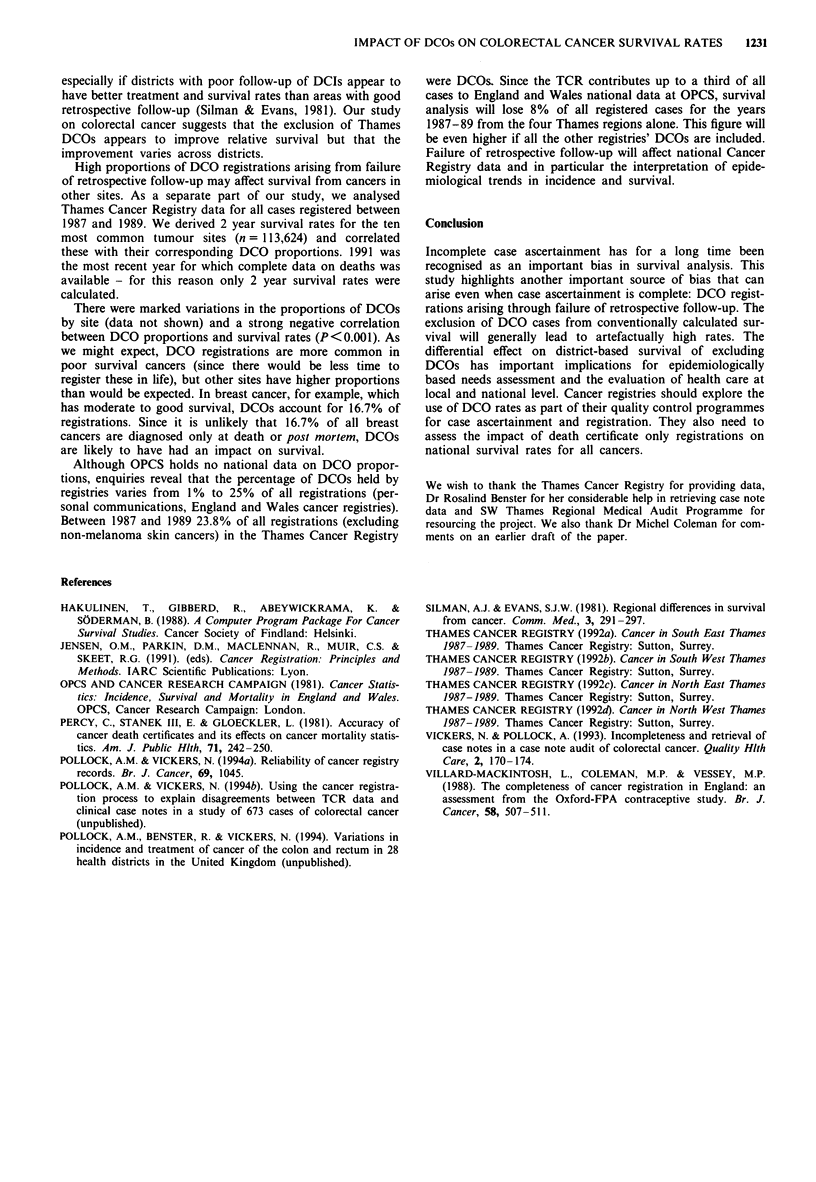# The impact on colorectal cancer survival of cases registered by 'death certificate only': implications for national survival rates.

**DOI:** 10.1038/bjc.1994.478

**Published:** 1994-12

**Authors:** A. M. Pollock, N. Vickers

**Affiliations:** Department of Public Health Sciences, St George's Hospital Medical School, London, UK.

## Abstract

This paper describes the effect of including death certificate only (DCO) registrations on 5 year relative survival rates for colorectal cancer in four district health authorities (DHAs) in south-east England. A retrospective case note study was set up to examine all cases of colorectal cancer listed in the Thames Cancer Registry (TCR) as having been diagnosed in 1983 and 1988 and resident in one of four districts, A, B, C and D. A total of 673 sets of cases notes were requested from all hospitals within the four districts, including 150 sets on DCO cases. Of 465 (69%) sets of case notes retrieved, 378 (72.3%) were non-DCO cases. Of these, 14 were excluded from survival analysis because of missing dates of diagnosis or death in the notes. Eighty-seven (58.0%) sets of case notes were retrieved on DCO registrations, of which seven were excluded because no date of diagnosis was available in the notes. Retrieval rates on case note registrations varied by DHA of residence: 73.3% in DHA A, 96.6% in DHA B, 34.5% in DHA C and 79.2% in DHA D. The corresponding figures for DCO registrations were 63.5%, 69.0%, 7.4% and 76.2%. Cumulative relative 5 year survival rates by DHA of residence were calculated first for cases registered from case notes and then for all cases including those registered solely from a death certificate. The total number of cases used in the survival analysis was 444 (18% DCOs). In all four DHAs, 5 year survival decreased with the inclusion of DCO registrations: by 9.1% in district A (from 52.8 to 43.7), by 4.5% in district B (from 59.6 to 55.1), by 4.8% in district C (from 80.0 to 75.2) and by 7.6% in district D (from 31.4 to 23.8). The overall reduction in survival was 8.6%. The exclusion of death certificate only registrations from survival data is an important source of bias. Using TCR data, we compared DCO proportions for colorectal cancer with other sites. DCO proportions were shown to vary by tumour site and survival time. The DCO registration is an important quality measure of ascertainment and follow-up. OPCS should publish DCO proportions by registry area and cancer site. Registries should implement DCO monitoring as part of quality improvement programmes.


Br. J. Cancer (1994), 70, 1229-1231 © Macmillan Press Ltd., 1994

### The impact on colorectal cancer survival of cases registered by 'death certificate only': implications for national survival rates

A.M. Pollock & N. Vickers

*Department of Public Health Sciences, St George's Hospital Medical School, Cranmer Terrace, London SW17 0RE, UK.*

**Summary** This paper describes the effect of including 'death certificate only' (DCO) registrations on 5 year relative survival rates for colorectal cancer in four district health authorities (DHAs) in south-east England. A retrospective case note study was set up to examine all cases of colorectal cancer listed in the Thames Cancer Registry (TCR) as having been diagnosed in 1983 and 1988 and resident in one of four districts, A, B, C and D. A total of 673 sets of case notes were requested from all hospitals within the four districts, including 150 sets on DCO cases. Of 465 (69%) sets of case notes retrieved, 378 (72.3%) were non-DCO cases. Of these, 14 were excluded from survival analysis because of missing dates of diagnosis or death in the notes. Eighty-seven (58.0%) sets of case notes were retrieved on DCO registrations, of which seven were excluded because no date of diagnosis was available in the notes. Retrieval rates on case note registrations varied by DHA of residence: 73.3% in DHA A, 96.6% in DHA B, 34.5% in DHA C and 79.2% in DHA D. The corresponding figures for DCO registrations were 63.5%, 69.0%, 7.4% and 76.2%. Cumulative relative 5 year survival rates by DHA of residence were calculated first for cases registered from case notes and then for all cases including those registered solely from a death certificate. The total number of cases used in the survival analysis was 444 (18% DCOs). In all four DHAs, 5 year survival decreased with the inclusion of DCO registrations: by 9.1% in district A (from 52.8 to 43.7), by 4.5% in district B (from 59.6 to 55.1), by 4.8% in district C (from 80.0 to 75.2) and by 7.6% in district D (from 31.4 to 23.8). The overall reduction in survival was 8.6%. The exclusion of death certificate only registrations from survival data is an important source of bias. Using TCR data, we compared DCO proportions for colorectal cancer with other sites. DCO proportions were shown to vary by tumour site and survival time. The DCO registration is an important quality measure of ascertainment and follow-up. OPCS should publish DCO proportions by registry area and cancer site. Registries should implement DCO monitoring as part of quality improvement programmes.

The Office of Population Censuses and Surveys (OPCS) publishes national 5 year cancer survival statistics, derived from returns made by the 12 cancer registries in England and Wales (OPCS and Cancer Research Campaign, 1981). The accuracy of these statistics depends on the completeness of case ascertainment by the registries and on the completeness and accuracy of the data sources from which registrations are made. Although registration is voluntary, ascertainment is estimated to be around 90% for the country as a whole (Villard-Mackintosh *et al.*, 1988). The main data sources for cancer registration are clinical case notes and copies of death certificates forwarded by OPCS to each registry on every person dying on its territory for whom cancer is mentioned.

Death certificate notification enables registries to identify cases not registered in life. At Thames Cancer Registry (TCR), the focus of this report, around 50% of the cases identified by death certificate will have already been registered. For the remainder, the death certificate is the first evidence obtained by a registry of the case and is used to initiate a new registration, termed a death certificate-initiated registration (DCI) (Jensen *et al.*, 1991; Thames Cancer Registry, 1992*a–d*). If, on the basis of death certificate information, a place of treatment can be traced, the registry attempts to obtain confirmation of the diagnosis from the hospitals nearest to the place of death, the certifying physician or coroner. This process is called retrospective follow-up. If no further information is obtained on a DCI registration during the follow-up period (6 months), the case is deemed a death certificate only registration (DCO) (Jensen *et al.*, 1991).

DCO registrations are important for two reasons. First, high proportions of DCOs cast doubt on the accuracy of cancer registry incidence data: Percy *et al.* (1981) reported only 65% accuracy in the coding of cause of death in death certificates on cancer patients. Second, because it is not usually possible to confirm the date of diagnosis for a DCO registration, they are excluded from cancer registry and OPCS survival analysis.

Historically, DCO registrations were based only on those cases that remained unregistered after intensive and extended searches for information on tumour site and date of diagnosis. Since 1983, a rapid increase has taken place in Thames DCO rates. The registry has explained this rise by reference to the decision taken in 1983 (for financial reasons) not to follow up patients dying at home (Thames Cancer Registry, 1992*a*). Since 1992, it has been attempting to retrieve data on DCO cases (including those patients dying at home) through Family Health Services Authorities (FHSAs).

A second reason for the high rate was the amalgamation of the North Thames Regions, which only became part of the territory covered by the TCR in 1985: the greatest concentration of DCOs was found in North-East Thames Region.

In 1990 in an ecological analysis of all cases of colorectal cancer registered by the Thames Cancer Registry and diagnosed between 1982 and 1988, we showed significant variations in 5 year survival rates and DCO proportions across the 28 districts in the two south Thames regions (Pollock *et al.*, 1994). To investigate the underlying reasons for these differences, we undertook a retrospective case note study in four of those districts (the two with the best survival and the two with the worst survival) (Vickers & Pollock, 1993; Pollock & Vickers, 1994*a*). In order to ascertain the extent to which the observed survival differences were artefactual (the result of differences in retrospective follow-up of DCI registrations), we also requested notes on all DCO registrations.

This paper examines the effect on 5 year survival rates of including cases registered by DCO for which it *was* possible to retrieve case notes and discusses the implications of our findings for national cancer survival statistics and the use of cancer registries for epidemiological and health services research.

### Methods

Between 1991 and 1993 we requested case notes on all cases which were diagnosed in 1983 and 1988 and resident in the

Correspondence: A.M. Pollock.
Received 22 February 1994; and in revised form 6 July 1994.

four districts identified *and* which were diagnosed or treated in their district of residence. Most patients are diagnosed and treated in their district of residence (83%, 88%, 73% and 79% in districts A, B, C and D respectively) (Vickers & Pollock, 1993). Case notes were requested from five medical record sites covering six NHS hospitals and outlying out-patient departments in the four districts.

Relative survival is the ratio of the survival observed in a group of cancer patients to the survival expected if they were only subject to the general (all-cause) mortality in a standard population. Using the Hakulinen computer program (Hakulinen *et al.*, 1988), we calculated cumulative 5 year relative survival rates by DHA of residence for (a) non-DCO cases, to obtain conventional survival rates, and (b) all cases including DCO cases for which we were able to ascertain a date of diagnosis from case notes. (Note that both analyses include only patients treated in their district of residence.)

### Results

Of the 673 cases identified from the TCR, 150 were DCO registrations (i.e. DCOs accounted for 22.3% of the total sample). A total of 465 (69.0%) sets of case notes were retrieved. Three hundred and seventy-eight sets of case notes were retrieved for non-DCO cases, 14 of which had to be excluded from survival analysis because of missing dates of diagnosis or death. Eighty-seven sets of case notes were retrieved for DCO registrations, seven of which had to be excluded because no date of diagnosis was listed in the notes. None of the DCOs for which case notes were found were diagnosed on the date they died.

Table I shows that retrieval rates on case note registrations varied by DHA of residence: 73.3% in DHA A, 96.6% in DHA B, 34.5% in DHA C and 79.2% in DHA D and 72.3% overall. The corresponding figures for DCO registrations were 63.5%, 69.0%, 7.4%, 76.2% and 58.0%.

The impact of DCOs on survival in all four DHAs combined is shown in Figure 1. Five year survival rates for colorectal cancer by the conventional method (excluding DCO cases) was 49.3%; when retrieved DCO cases were included survival rates fell to 40.9%. The 5 year survival rate

**Table I** Retrieval rates by district of residence and source of registration

| DHA | Case note registrations n | Case note registrations % | DCO registrations n | DCO registrations % |
|---|---|---|---|---|
| A | 121/165 | 73.3 | 33/52 | 63.5 |
| B | 141/146 | 96.6 | 20/29 | 69.0 |
| C | 40/116 | 34.5 | 2/27 | 7.4 |
| D | 76/96 | 79.2 | 32/42 | 76.2 |
| Total | 378/523 | 72.3 | 87/150 | 58.0 |

**Figure 1** The impact of DCO cases on colorectal cancer 5 year relative survival rate (RSR) ($n = 444$). O, Excluding DCOs; □, including DCOs; Δ, DCOs.

for the DCO group only was 7.3%. (DCOs constituted 18.0% of the 444 cases included in the survival analysis.)

The impact of DCOs on each of the four districts survival rates is shown in Table II. In all four DHAs, 5 year survival decreased with the inclusion of DCO registrations, district A by 9.1% (from 52.8 to 43.7), by 4.5% in district B (from 59.6 to 55.1), by 4.8% in district C (from 80.0 to 75.2) and 7.6% in district D (from 31.4 to 23.8). The greatest survival differences occurred during the first year from diagnosis. District D, which had the highest proportion of DCOs and the best DCO retrieval rate, had the largest decrease in 1 year relative survival (9.6%).

### Discussion

Our results suggest that conventionally derived cancer survival rates can be artefactually high, because of exclusion of cases registered only from a death certificate: these cases typically have very short survival times. Our original sample included 22.3% DCOs. Notes were retrieved on 58.0% of these, and DCOs accounted for 18% of the records analysed for survival. Overall 5 year relative survival fell from 49.3% to 40.9% when DCOs were included. Most of the survival difference occurred during the first year from diagnosis and was carried forward in subsequent years. This might be expected given that the probability of registration from case notes should increase with longer survival time (Pollock & Vickers, 1994*b*).

The retrieval rate of 72.3% for non-DCO cases is lower than the rate we had hoped to achieve: this was partly because 108 (29.6%) cases had been diagnosed in 1983, i.e. 10 years before case notes were requested, and partly because of differences in the operation of medical records departments across the four districts (Vickers & Pollock, 1993). Because of the possibility of selection bias in the unretrieved cases, we did not carry out significance tests on the two sets of relative survival rates (including and excluding cases registered by DCO).

Five year survival rates fell by between 4.5% and 9.1% across the four districts when DCO cases were included. We could not establish how much of this variation was due to differences in case note retrieval on DCOs across districts, and how much to differences in the proportion of DCOs across districts. Nonetheless, this result has important implications for health services research and evaluation,

**Table II** Comparison of cumulative relative 5 year survival rates (%) for all cases in the conventional analysis (i.e. excluding DCOs) and all cases including DCOs by DHA of residence

| DHA | Years | Conventional analysis % | Conventional analysis n | Including traced DCO cases % | Including traced DCO cases n |
|---|---|---|---|---|---|
| A | 1 | 68.2 | 119 | 62.1 | 150 |
| | 2 | 52.5 | 77 | 48.7 | 88 |
| | 3 | 48.7 | 56 | 42.2 | 65 |
| | 4 | 49.6 | 49 | 42.4 | 53 |
| | 5 | 52.8 | 47 | 43.7 | 50 |
| B | 1 | 82.4 | 134 | 78.1 | 153 |
| | 2 | 68.2 | 105 | 63.3 | 113 |
| | 3 | 63.2 | 82 | 59.3 | 86 |
| | 4 | 60.3 | 72 | 56.4 | 76 |
| | 5 | 59.6 | 65 | 55.1 | 68 |
| C | 1 | 90.7 | 36 | 88.6 | 38 |
| | 2 | 80.2 | 31 | 78.7 | 32 |
| | 3 | 71.5 | 26 | 67.5 | 27 |
| | 4 | 75.5 | 22 | 71.1 | 22 |
| | 5 | 80.0 | 22 | 75.2 | 22 |
| D | 1 | 54.1 | 76 | 44.5 | 103 |
| | 2 | 42.8 | 39 | 33.2 | 43 |
| | 3 | 32.6 | 29 | 25.9 | 30 |
| | 4 | 29.5 | 21 | 23.9 | 22 |
| | 5 | 31.4 | 18 | 23.8 | 19 |

especially if districts with poor follow-up of DCIs appear to have better treatment and survival rates than areas with good retrospective follow-up (Silman & Evans, 1981). Our study on colorectal cancer suggests that the exclusion of Thames DCOs appears to improve relative survival but that the improvement varies across districts.

High proportions of DCO registrations arising from failure of retrospective follow-up may affect survival from cancers in other sites. As a separate part of our study, we analysed Thames Cancer Registry data for all cases registered between 1987 and 1989. We derived 2 year survival rates for the ten most common tumour sites ($n = 113,624$) and correlated these with their corresponding DCO proportions. 1991 was the most recent year for which complete data on deaths was available – for this reason only 2 year survival rates were calculated.

There were marked variations in the proportions of DCOs by site (data not shown) and a strong negative correlation between DCO proportions and survival rates ($P < 0.001$). As we might expect, DCO registrations are more common in poor survival cancers (since there would be less time to register these in life), but other sites have higher proportions than would be expected. In breast cancer, for example, which has moderate to good survival, DCOs account for 16.7% of registrations. Since it is unlikely that 16.7% of all breast cancers are diagnosed only at death or *post mortem*, DCOs are likely to have had an impact on survival.

Although OPCS holds no national data on DCO proportions, enquiries reveal that the percentage of DCOs held by registries varies from 1% to 25% of all registrations (personal communications, England and Wales cancer registries). Between 1987 and 1989 23.8% of all registrations (excluding non-melanoma skin cancers) in the Thames Cancer Registry were DCOs. Since the TCR contributes up to a third of all cases to England and Wales national data at OPCS, survival analysis will lose 8% of all registered cases for the years 1987–89 from the four Thames regions alone. This figure will be even higher if all the other registries' DCOs are included. Failure of retrospective follow-up will affect national Cancer Registry data and in particular the interpretation of epidemiological trends in incidence and survival.

### Conclusion

Incomplete case ascertainment has for a long time been recognised as an important bias in survival analysis. This study highlights another important source of bias that can arise even when case ascertainment is complete: DCO registrations arising through failure of retrospective follow-up. The exclusion of DCO cases from conventionally calculated survival will generally lead to artefactually high rates. The differential effect on district-based survival of excluding DCOs has important implications for epidemiologically based needs assessment and the evaluation of health care at local and national level. Cancer registries should explore the use of DCO rates as part of their quality control programmes for case ascertainment and registration. They also need to assess the impact of death certificate only registrations on national survival rates for all cancers.

We wish to thank the Thames Cancer Registry for providing data, Dr Rosalind Benster for her considerable help in retrieving case note data and SW Thames Regional Medical Audit Programme for resourcing the project. We also thank Dr Michel Coleman for comments on an earlier draft of the paper.

### References

HAKULINEN, T., GIBBERD, R., ABEYWICKRAMA, K. & SÖDERMAN, B. (1988). *A Computer Program Package For Cancer Survival Studies*. Cancer Society of Findland: Helsinki.

JENSEN, O.M., PARKIN, D.M., MACLENNAN, R., MUIR, C.S. & SKEET, R.G. (1991). (eds). *Cancer Registration: Principles and Methods*. IARC Scientific Publications: Lyon.

OPCS AND CANCER RESEARCH CAMPAIGN (1981). *Cancer Statistics: Incidence, Survival and Mortality in England and Wales*. OPCS, Cancer Research Campaign: London.

PERCY, C., STANEK III, E. & GLOECKLER, L. (1981). Accuracy of cancer death certificates and its effects on cancer mortality statistics. *Am. J. Public Hlth*, **71**, 242–250.

POLLOCK, A.M. & VICKERS, N. (1994*a*). Reliability of cancer registry records. *Br. J. Cancer*, **69**, 1045.

POLLOCK, A.M. & VICKERS, N. (1994*b*). Using the cancer registration process to explain disagreements between TCR data and clinical case notes in a study of 673 cases of colorectal cancer (unpublished).

POLLOCK, A.M., BENSTER, R. & VICKERS, N. (1994). Variations in incidence and treatment of cancer of the colon and rectum in 28 health districts in the United Kingdom (unpublished).

SILMAN, A.J. & EVANS, S.J.W. (1981). Regional differences in survival from cancer. *Comm. Med.*, **3**, 291–297.

THAMES CANCER REGISTRY (1992*a*). *Cancer in South East Thames 1987–1989*. Thames Cancer Registry: Sutton, Surrey.

THAMES CANCER REGISTRY (1992*b*). *Cancer in South West Thames 1987–1989*. Thames Cancer Registry: Sutton, Surrey.

THAMES CANCER REGISTRY (1992*c*). *Cancer in North East Thames 1987–1989*. Thames Cancer Registry: Sutton, Surrey.

THAMES CANCER REGISTRY (1992*d*). *Cancer in North West Thames 1987–1989*. Thames Cancer Registry: Sutton, Surrey.

VICKERS, N. & POLLOCK, A. (1993). Incompleteness and retrieval of case notes in a case note audit of colorectal cancer. *Quality Hlth Care*, **2**, 170–174.

VILLARD-MACKINTOSH, L., COLEMAN, M.P. & VESSEY, M.P. (1988). The completeness of cancer registration in England: an assessment from the Oxford-FPA contraceptive study. *Br. J. Cancer*, **58**, 507–511.